# *TMPRSS2:ERG* gene fusion variants induce TGF-β signaling and epithelial to mesenchymal transition in human prostate cancer cells

**DOI:** 10.18632/oncotarget.15931

**Published:** 2017-03-06

**Authors:** Leonie Ratz, Mark Laible, Lukasz A Kacprzyk, Stephanie M Wittig-Blaich, Yanis Tolstov, Stefan Duensing, Peter Altevogt, Sabine M Klauck, Holger Sültmann

**Affiliations:** ^1^ Cancer Genome Research Group, German Cancer Research Center (DKFZ), German Cancer Consortium (DKTK), and National Center for Tumor Diseases (NCT), 69120 Heidelberg, Germany; ^2^ Department of Urology, Section of Molecular Urooncology, University Hospital Heidelberg, 69120 Heidelberg, Germany; ^3^ Skin Cancer Unit, German Cancer Research Center (DKFZ), 69120 Heidelberg, Germany; ^4^ Department of Dermatology, Venereology and Allergology, University Medical Center Mannheim, Ruprecht-Karls University of Heidelberg, 68135 Mannheim, Germany; ^5^ Present address: BioNTech Diagnostics GmbH, 55131 Mainz, Germany; ^6^ Present address: UGA Biopharma GmbH, 16761 Hennigsdorf, Germany; ^7^ Present address: Institute of Comparative Molecular Endocrinology (CME), University of Ulm, 89081 Ulm, Germany

**Keywords:** TMPRSS2:ERG fusion variants, TGF-β signaling, ALK1, EMT, prostate cancer

## Abstract

*TMPRSS2:ERG* (T/E) gene fusions are present in approximately 50% of all prostate cancer (PCa) cases. The expression of fusion mRNAs from distinct T/E variants is associated with clinicopathological parameters, while the underlying molecular processes remain unclear. We characterized the molecular mechanisms and functional implications caused by doxycycline (Dox)-inducible overexpression of the frequent T/E III and VI fusion variants in LNCaP cells. Induction of T/E expression resulted in increased cellular migratory and invasive potential, and reduced proliferation and accumulation in G1 phase. T/E overexpressing cells showed epithelial-to-mesenchymal transition (EMT), as demonstrated by upregulation of TGF-β and WNT pathway genes, mesenchymal markers, and increased phosphorylation of the p38 MAPK. Augmented secretion of TGF-β1 and –β2, and T/E-mediated regulation of ALK1, a member of the TGF-β receptor family, was detected. *ALK1* inhibition in T/E overexpressing cells blocked p38 phosphorylation and reduced the expression of the TGF-β target genes *VIM*, *MMP1*, *CDH2*, and *SNAI2*. We found a T/E variant VI-specific induction of *miR-503* associated with reduced expression of SMAD7 and CDH1. Overexpression of *miR-503* led to increased levels of *VIM* and *MMP1*. Our findings indicate that TGF-β signaling is a major determinant of EMT in T/E overexpressing LNCaP cells. We provide evidence that T/E VI-specific transcriptional modulation by miR-503 accounts for differences in the activation of EMT pathway genes, promoting the aggressive phenotype of tumors expressing T/E variant VI. We suggest that ALK1-mediated TGF-β signaling is a novel oncogenic mechanism in T/E positive PCa.

## INTRODUCTION

Prostate cancer (PCa) is the most frequently diagnosed cancer among men in Western countries and a major cause of cancer-related mortality [1, 2]. PCa is a heterogeneous disease with several molecular and clinicopathological subtypes. The *TMPRSS2:ERG* (T/E) gene fusion, resulting from a chromosomal rearrangement of *ERG* (v-ets erythroblastosis virus E26 homolog (avian)) to the androgen responsive gene *TMPRSS2* (transmembrane protease, serine 2), is the most frequent somatic alteration in PCa [3], and detectable in 50% of the tumors [4]. In those cases, *ERG* overexpression is driven by the androgen-responsive promoter of *TMPRSS2*, resulting in upregulation of ERG protein and activation of downstream target genes [5]. Ninety percent of PCas overexpressing ERG harbor T/E fusions [5]. However, no consensus on the prognostic significance of T/E fusion-positive tumors has been reached so far [6, 7]. This may be due to differences in tumor characteristics and multiple T/E isoforms [6, 8, 9], which have been associated with clinicopathological parameters [10] and progression [11, 12]. The most common fusion mRNA variant III (T/E III), containing exon 1 of *TMPRSS2* (1-17bp) and exons 4-11 of *ERG* (T1/E4), is present in 86% of fusion-positive tumors [10]. Since exon 1 of *TMPRSS2* is noncoding, this mRNA is translated from an internal ATG site, resulting in a truncated ERG protein. The expression of T/E VI, resulting from fusion of exons 1-2 of *TMPRSS2* to exons 4-11 of *ERG* (T2/E4), has been associated with aggressive disease [10]. This mRNA is translated from a start codon within *TMPRSS2* exon 2 that is in frame with the *ERG* ORF. The resulting protein includes the first five amino acids of TMPRSS2 and lacks the first 12 amino acids of the full-length ERG protein.

Previously, we found T/E specific transcriptional upregulation of genes associated with activated TGF-β/BMP and WNT signaling in fusion-positive PCa compared to fusion-negative PCa [13]. TGF-β and WNT signaling regulate a diverse range of cellular processes related to cancer progression [14, 15] and are major inducers of epithelial-to-mesenchymal transition (EMT) [16]. Here, our aim was to characterize the molecular mechanisms and functional implications of T/E variant overexpression and their consequences on cellular and molecular phenotypes. We focused on the analysis of T/E III and T/E VI gene fusion variants based on their frequencies of occurrence and their association with clinical and pathological variables. We established LNCaP cells, featuring androgen-independency with high levels of androgen receptor (AR), stably overexpressing the T/E III and VI variants in an inducible promoter system (LNCaP-T/E) and examined the effects of overexpression on cellular properties and signal transduction pathways. To validate the observed transcriptional modulation upon ERG overexpression in LNCaP, the T/E-positive prostate cancer cell line NCI-H660 [17] was employed. This cell line harbors both T/E III and T/E VI fusions [17]. Complementary to the LNCaP-T/E model, ERG was silenced in NCI-H660 using an ERG-specific siRNA and mRNA levels of the targets previously measured in LNCaP-T/E clones were assessed. Overall, we found a large degree of commonality but also distinct transcriptional effects between T/E III and VI variants.

## RESULTS

### Characterization of T/E expressing LNCaP cells

**Figure 1:**
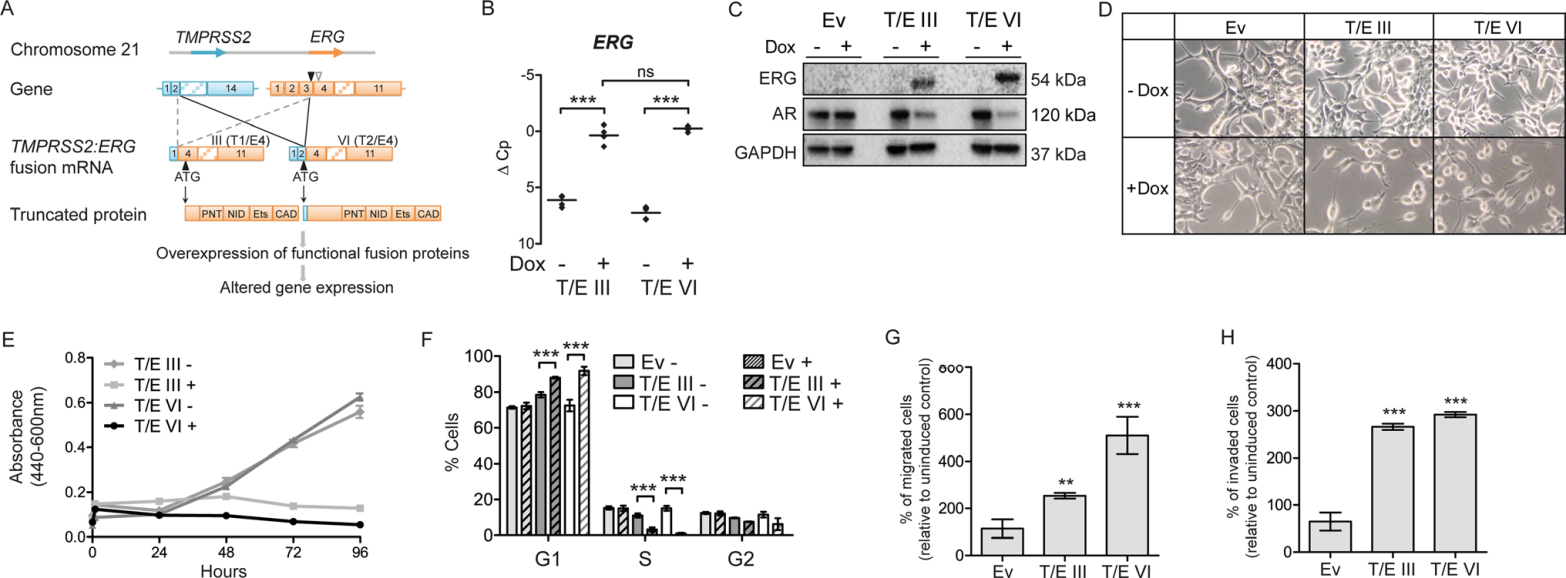
S/E variant overexpression in LNCaP cells **(A)** Structure of T/E gene fusion variants III and VI; *TMPRSS2* (RefSeq NM_005656), *ERG* (RefSeq NM_004449.4). Downward pointing arrowheads: position of *ERG* fusion break point in T/E III (white) and T/E VI (black). Upward black pointing arrowhead: translation initiation codon. Protein domains: PNT, pointed domain (a protein–protein interaction site); NID, N-terminal inhibitory domain; Ets, Ets-DNA binding domain; CAD, C-terminal activator domain. **(B)** qPCR of Dox-induced *ERG* expression in T/E III and T/E VI compared to uninduced cells. ΔC *p* values from three independent experiments are shown relative to *GAPDH*. **(C)** Western blot analysis of ERG and AR expression in empty vector (Ev), T/E III and T/E VI LNCaP cells, respectively. GAPDH served as protein loading control. **(D)** Morphological changes induced by Dox-mediated ERG overexpression in T/E III and T/E VI and control LNCaP cells. Pictures were taken at 20-fold magnification after 72h Dox induction. **(E)** Cell growth was measured by a WST-1 assay at the indicated time points after pre-treatment with Dox (three independent experiments). **(F)** Cell cycle analysis using flow cytometry with propidium iodide (PI) staining. T/E III, T/E VI and empty vector cells were either not treated (−) or treated with Dox (+) and analyzed 72h post induction. Data are shown as percent positive staining cells ± s.d. of three independent experiments. **(G–H)** Quantification of (G) migrated, and (H) invaded T/E expressing cells with a transwell chamber assay. Eight microscopic fields per treatment were analyzed and results of three independent experiments are shown. Ev – Empty vector; ns – not significant.

To study the role of the T/E gene fusion variants (Figure 1A), we made use of a Flp recombinase based transfection system allowing stable and inducible expression of T/E variants III and VI in LNCaP cells. An empty expression vector served as a control. The expression of T/E variants was verified using RT-PCR (Supplementary Figure 1B). QPCR analysis after Dox-induction showed ∼50-fold and ∼150-fold upregulation of *ERG* in T/E III and T/E VI cells, respectively (Figure 1B). Western blot analysis confirmed the expression of ERG protein in Dox-induced LNCaP-T/E cells only (Figure 1C). In line with previous reports that ERG expression leads to downregulation of *AR* transcripts [18], both LNCaP-T/E III and VI cell lines showed markedly decreased AR protein after ERG overexpression (Figure 1C), indicating that the cell lines faithfully reflect the *in vivo* situation. Concurrent with reports that lower AR expression is associated with reduced differentiation of PCa cells [19], we noticed morphological changes, including cellular rounding, spindle-like branching, and detachment from adjacent cells (Figure 1D), which resembled a fibroblast-like morphology. These results suggested that ERG affects processes controlling the morphology of LNCaP cells.

### T/E overexpression confers oncogenic properties to LNCaP cells

The impact of T/E overexpression on LNCaP cells was analyzed using proliferation, migration and invasion assays. T/E overexpressing cells showed reduced proliferation from 48 h to 96 h post induction (Figure 1E). After 72 h, a decreased number of cells in S- and G2-phase while an increased number in G1-phase was observed (Figure 1F) for both T/E III and VI variants. No apoptotic cells were detectable in the sub-G1 fractions. Thus, T/E overexpression induced cell cycle changes leading to the accumulation of cells in G1 phase and reduced cell proliferation without apoptosis. T/E expressing LNCaP cells migrated and invaded significantly faster compared to uninduced or empty vector controls (Figure 1G and 1H) and the migration rate was higher in T/E VI compared to T/E III cells (Figure 1G).

### Overexpression of T/E III and VI variants reveal transcriptional programs associated with TGF-β signaling

The transcriptional programs regulated by T/E overexpression were investigated by microarray expression profiling on 48,107 genes (GEO accession GSE78032). Differentially expressed genes (> 1.5 fold change cut-off; *p* < 0.05) compared to empty vector control (*n* = 4,429; Supplementary Table 1) were selected for further analysis using the Ingenuity Pathway Analysis (IPA) program. Of the 2,205 genes, which were altered in both T/E III and VI variants (*T/E intersection*; Supplementary Table 1), 94% showed concordant expression changes, indicating a high degree of accordance between the variants. The number of distinct genes found after T/E III or VI overexpression (*T/E III only* and *T/E VI only*) was 418 and 1,806, respectively (Supplementary Table 1).

Comparison of differential mRNA expression between LNCaP-T/E cells and T/E-positive *ex vivo* tumors [13] revealed 30% (37/126) overlap, including the genes Tudor Domain Containing 1 *(TDRD1*), Cluster of Differentiation 24 (*CD24*), BMP And Activin Membrane-Bound Inhibitor (*BAMBI*), and Cyclin-Dependent Kinase 1 (*CDK1*) [13]. Furthermore, transcriptional changes in T/E overexpressing cells were consistent with the expected transcriptional response to ERG overexpression based on previous findings. For example, *AR* and the androgen-responsive genes *TMPRSS2* [5, 18], *SLC45A3* [5, 18, 19], *ACPP* [19], and *MSMB* [5, 19] were downregulated, whereas genes known to be activated by ERG, e.g. *PLAT* [18], *PLA1A* [5], and *MMP1* [20] were upregulated. These data indicated that our T/E expressing cell models faithfully reflected the transcriptional regulation in T/E-positive tumors and are suio study the biology of the variants.

GO analysis in IPA using the *T/E intersection* (*n* = 2,205) showed that genes associated with cell proliferation and interphase were downregulated (Supplementary Table 2), which was in agreement with the reduced proliferative ability of LNCaP-T/E cells (Figure 1E). ‘Estrogen-mediated S-phase Entry’ was identified among the top significantly enriched canonical pathways (Supplementary Table 3). This corresponded to the accumulation of the T/E overexpressing cells in G1 phase (Figure 1F). Consistent with the increased number of migrated and invaded cells found in the transwell assays (Figure 1G and 1H), genes belonging to the category ‘Cell invasion’ were primarily upregulated (Supplementary Table 2).

**Figure 2:**
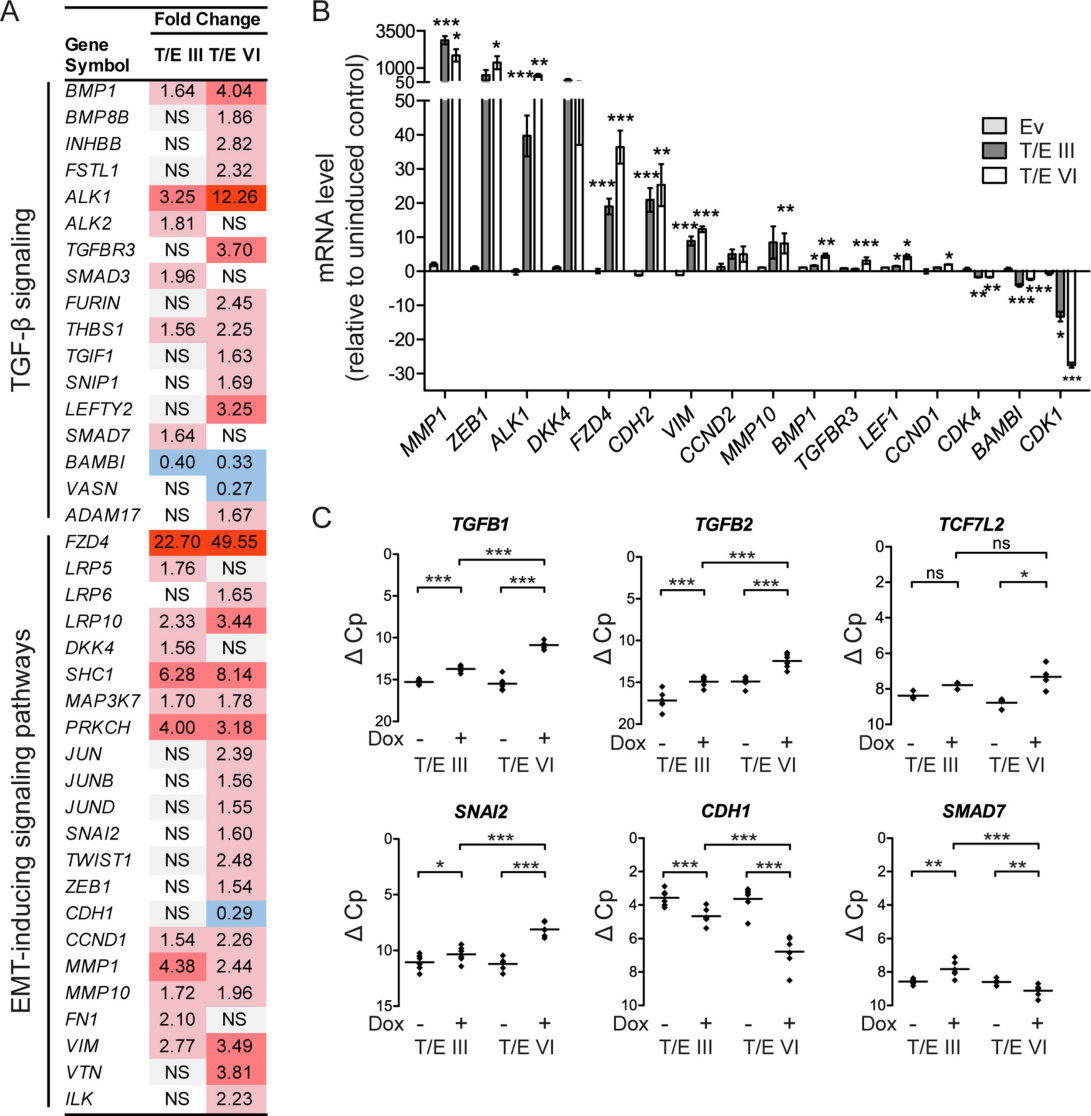
Transcriptional modulation in LNCaP-T/E cells **(A)** Microarray gene expression data indicating activated TGF-β and EMT-inducing signaling pathways in T/E expressing cells. **(B–C)** qPCR validation of T/E-induced gene expression changes associated with an EMT profile. (B) mRNA expression after T/E induction relative to uninduced controls. (C) mRNA levels of selected targets showing prominent transcriptional modulation in T/E III and VI overexpressing cells. Data are shown as ΔC *p* values relative to *GAPDH* measured in the same samples, which had been used for the microarray analysis. Ev - Empty vector; NS/ns - not significant. Red - upregulated; blue - downregulated.

To study potential mechanisms regulating transcriptional changes associated with increased migration and invasion in LNCaP-T/E cells, an upstream regulator analysis was performed with the *T/E intersection* dataset (Supplementary Table 1). This analysis revealed transforming growth factor beta 1 (*TGFB1*) as an upstream regulator of 284 genes that were directly associated with *TGFB1* regulation (Supplementary Table 4), and indicated that TGF-β signaling plays a crucial role in T/E overexpressing cells as previously suggested by us [13]. Evidence for activated TGF-β signaling in the differentially expressed gene set (*n* = 4,429) was provided by upregulation of several TGF-β pathway-specific genes, e.g. bone morphogenetic protein 1 (*BMP1*), or downregulation of negative regulators of TGF-β signaling, such as the pseudoreceptor *BAMBI* (Figure 2A). Activation of noncanonical TGF-β signaling was evident by upregulation of SMAD-independent molecules involved in the ERK, JNK/p38 and PI3K/AKT pathways (Figure 2A). We also found upregulation of EMT-inducing transcription factors (*SNAI2*, *ZEB1*), mesenchymal markers (*FN1*, *VIM*, *VTN*) and matrix metalloproteinases (*MMP1*, *MMP10*), and downregulation of E-cadherin (*CDH1*) (Figure 2A), again supporting our finding that T/E expressing cells lose epithelial characteristics and acquire a mesenchymal phenotype. Of note, the upregulated genes included the type I TGF-β receptor Activin A Receptor Like Type 1 (*ACVRL1*, also known as *ALK1*) (Figure 2A), as well as the WNT receptor Frizzled 4 (*FZD4*) and its co-receptors *LRP5* and *LRP6* (Figure 2A), suggesting that these receptors might be mediators of transcriptional changes leading to EMT in T/E overexpressing cells.

Validation of the microarray data by qPCR verified the strong upregulation of *ALK1* and *FZD4* in both LNCaP-T/E variants (Figure 2B) as well as upregulation of EMT-inducing components and downregulation of genes negatively associated with TGF-β signaling (Figure 2B). Notably, the validation confirmed the strong upregulation of the selected EMT inducing ligands (*TGFB1*, 7.3-fold; *TGFB2*, 5.5-fold) and downstream effector genes (*TCF7L2*, 5.1-fold; *SNAI2*, 4.7-fold) in T/E VI cells, whereas *CDH1* (4.3-fold) and *SMAD7* (2.3-fold) were downregulated in T/E VI compared to T/E III expressing cells (Figure 2C).

### Distinct intracellular signaling molecules are regulated in T/E variants

**Figure 3:**
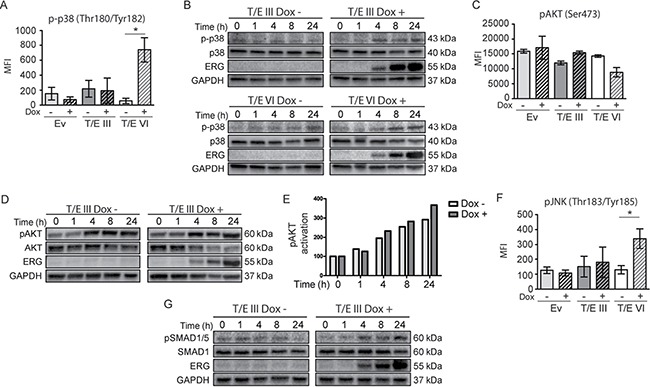
Signaling pathways in T/E expressing cells Activation of signaling molecules (p38, AKT, JNK, and SMAD1/5) in T/E expressing cells measured by Luminex technology **(A, C, F)**, and Western blot analysis **(B, D, G)**. For Western blot analysis, GAPDH served as loading control. Increased p38 phosphorylation (Thr180/Tyr182) was evident in T/E III (B) and T/E VI (A, B) cells. T/E III cells showed increased AKT phosphorylation (Ser473) (C, D). **(E)** pAKT/AKT ratios after densitometric analysis of Western blot bands of (D). (F) Increased JNK phosphorylation (Thr183/Tyr185) was revealed in T/E VI cells. Increased pSMAD1/5 phosphorylation (Ser463/465) was evident in (G) T/E III cells.

TGF-β signaling is mediated by SMAD-dependent and -independent signaling pathways involving JNK/p38 MAPK and PI3K/AKT [21, 22]. Multiplex protein quantification using Luminex technology and Western blot (Figure 3) for TGF-β signaling analysis revealed increased phosphorylation of p38 MAPK (Figure 3A and 3B), AKT (T/E III cells only; Figure 3C, 3D and 3E), JNK (Figure 3F), and SMAD1/5 (Figure 3G) upon T/E overexpression. Increased levels of p-SMAD1/5 and p-p38 were detectable 4h after T/E induction, concomitant with increasing ERG levels in T/E III and T/E VI cells. Increased phosphorylation of SMAD2 and 3 was not observed (data not shown). The increased AKT phosphorylation in T/E III, but not in T/E VI, expressing cells (Figure 3C, 3D and 3E) went along with the increasing ERG expression as confirmed by quantitative analysis showing the pAKT/AKT ratio in induced and uninduced cells, albeit this was not significant (Figure 3E). High basal levels of pAKT were observed in T/E III cells (Figure 3D). Activation of survival-associated processes corresponded to the functional annotation of the T/E gene set (Supplementary Table 2). TGF-β-mediated AKT activation has previously been proposed to overcome the growth-inhibitory effects of TGF-β in BPH1 tumorigenic sublines [23]. Taken together, these findings provided evidence for increased TGF-β signaling in both T/E variants, as well as an activated AKT dependent survival network upon T/E III overexpression, that might act together to induce EMT in this PCa cell model.

### Soluble TGF-β is produced by T/E overexpressing cells

**Figure 4:**
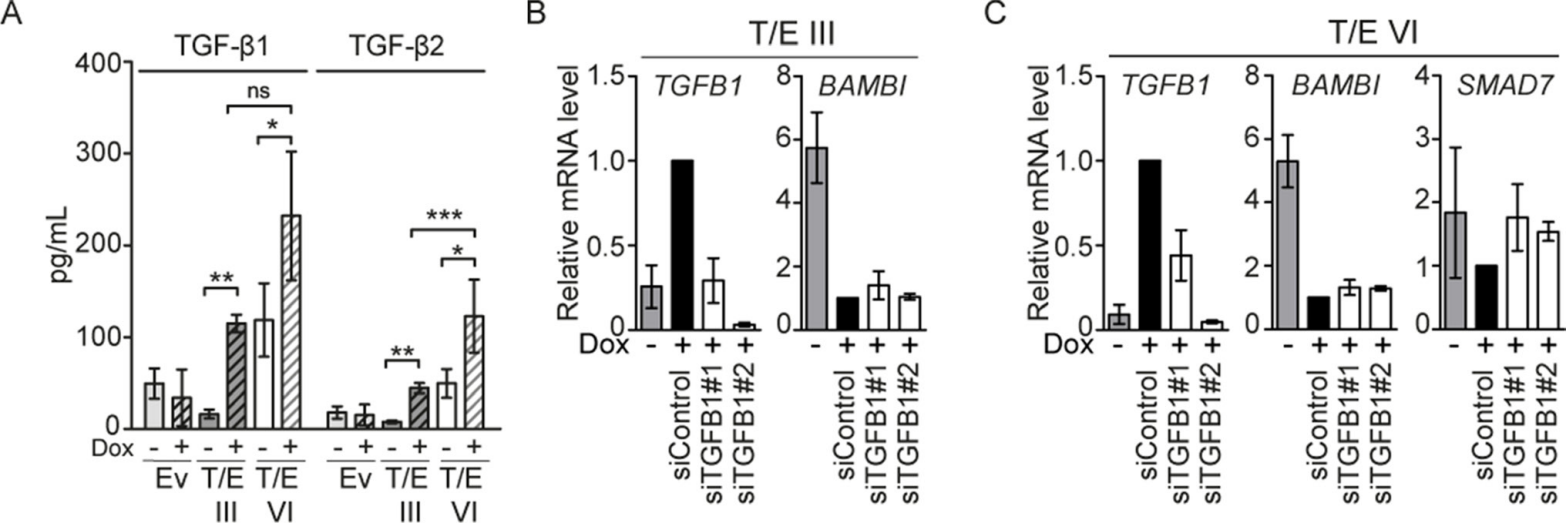
TGF-β signaling in LNCaP-T/E cells **(A)** TGF-β1 and TGF-β2 released into the medium was determined in cell-free conditioned medium derived from Dox-induced (+) and uninduced (−) LNCaP-T/E cells, respectively. All Luminex results are presented as MFI (mean fluorescent intensity) values ± s.d. of three independent experiments. Ev - Empty vector. **(B–C)** siRNA-mediated knockdown of *TGFB1* using 20 nM siRNA show upregulation of *BAMBI* in (B) T/E III and (C) T/E VI cells and additionally *SMAD7* in T/E VI cells (C) as determined by qPCR.

**Figure 5:**
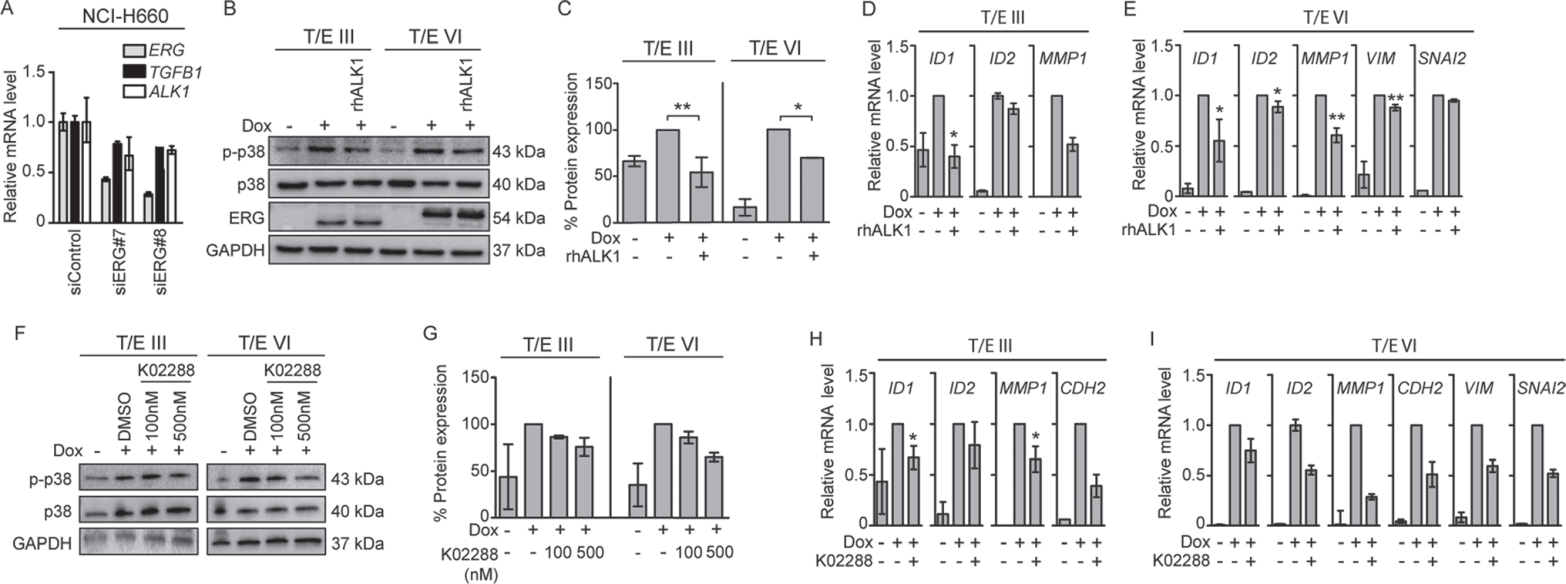
ALK1 inhibitors decrease T/E-induced ALK1 signaling **(A)** *ERG* knockdown in NCI-H660 cells (50 nM) showed reduced levels of *TGFB1* and *ALK1*. **(B)** Western blot analysis in LNCaP-T/E cells revealed reduced p38 phosphorylation after treatment with rhALK1 (5μg/mL) compared to PBS-treated control cells. **(C)** p-p38/p38 ratios after densitometric analysis of Western blot bands shown in (B) in T/E III and T/E VI cells, respectively. **(D–E)** Expression levels of TGF-β-responsive genes in (D) T/E III and (E) T/E VI expressing cells after rhALK1 treatment (5μg/mL) determined by qPCR. **(F)** Western blot analysis of p38 phosphorylation after treatment with K02288 (at indicated concentrations) compared to DMSO-treated control cells. **(G)** p-p38/p38 ratios after densitometric analysis of Western blot bands shown in (F) of T/E III and T/E VI cells, respectively. **(H–I)** Expression levels of TGF-β/BMP-responsive genes in (H) T/E III and (I) T/E VI expressing cells after simultaneous treatment with Dox and K02288 (500nM) were determined by qPCR.

Increased *TGFB1* and *-2* levels in T/E overexpressing cells motivated us to test whether TGF-β is secreted to act in an autocrine manner. Active TGF-β was measured in cell-free conditioned medium using Luminex immunoassays 72 h after Dox induction. T/E III and VI overexpressing cells displayed considerably increased TGF-β1 (7-fold and 2-fold, respectively) and TGF-β2 (6-fold and 3-fold, respectively) protein compared to controls (Figure 4A). Thus, T/E overexpression induced secretion of TGF-β1 and TGF-β2. SiRNA-mediated *ERG* knockdown in the NCI-H660 PCa cell line carrying both T/E fusion variants III and VI [17] reduced *TGFB1* mRNA levels (Figure 5A), further supporting the T/E-mediated upregulation of TGF-β ligands. Moreover, siRNA-mediated *TGFB1* knockdown in T/E III and VI cells led to upregulation of the negative TGF-β regulators *BAMBI* (in T/E III and VI cells) and *SMAD7* (only in T/E VI cells, Figure 4B and 4C), indicating that *TGFB1* plays a role in T/E-induced TGF-β signaling.

### ALK1 signaling regulates p38 MAPK and EMT markers

Since *ALK1* mRNA was strongly upregulated in T/E overexpressing cells (40-fold in T/E III and 500-fold in T/E VI cells; Figure 2B), we analyzed this pathway in more detail. *ERG* knockdown in NCI-H660 cells led to reduced *ALK1* mRNA levels (Figure 5A) confirming the association between ERG and ALK1 expression. Next, Dox-induced LNCaP-T/E cells were incubated with a human recombinant decoy receptor (rhALK1) [24] or the ALK1 inhibitor K02288 [25]. Disruption of ALK1 signaling using rhALK1 (Figure 5B and 5C) or K02288 (Figure 5F and 5G) resulted in reduced p38 phosphorylation. As expected [26, 27], the inhibitor of differentiation 1 (*ID1*), and *ID2* genes were upregulated after ERG induction, but reduced after rhALK1 (Figure 5D and 5E) or K02288 (Figure 5H and 5I) treatment. These data suggested that T/E overexpression induces ALK1-signaling and supported the concept that ALK1-mediated phosphorylation of p38 confers mesenchymal transformation of PCa cells. In line with this, rhALK1-mediated inhibition of ALK1 signaling led to reduced expression of *MMP1* (52% reduction) in T/E III (Figure 5D), and *MMP1*, *VIM*, and *SNAI2* (39%, 22%, and 5% reduction, respectively) in T/E VI cells (Figure 5E). ALK1 inhibition by K02288 also resulted in reduced expression of *MMP1* and *CDH2* (35% and 61% reduction, respectively) in T/E III (Figure 5H) and *MMP1*, *CDH2*, *VIM*, and *SNAI2* (72%, 50%, 40%, and 48% reduction, respectively) in T/E VI overexpressing cells (Figure 5I).

### T/E overexpression activates β-catenin signaling in prostate cancer cells

**Figure 6:**
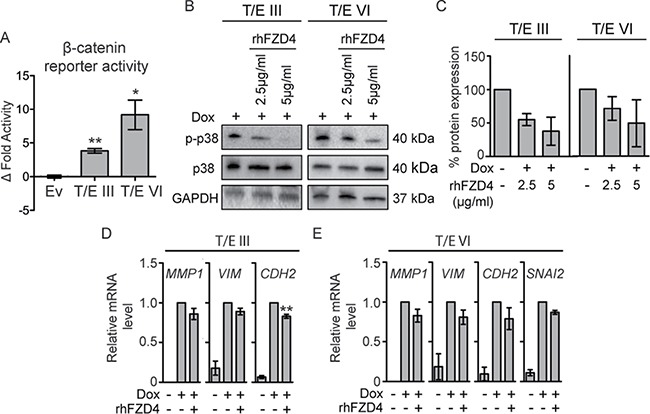
T/E expression induces FZD4-mediated β-catenin signaling in LNCaP cells **(A)** TOPflash luciferase activity 72 h post induction. Mean ± s.d. of three independent experiments are shown. TOPflash activity was normalized to mutant FOPflash activity and relative to uninduced clones. (**B**–**E**) Effects of FZD4-specific inhibition using rhFZD4 for 48h compared to PBS-treated control cells. (B) Phosphorylation of p38 was measured by Western blotting and p-p38/p38 (C) ratio was determined after band analysis of (B). (D–E) EMT target gene expression after rhFZD4 treatment (5μg/ml) was assessed by qPCR in T/E III (D) and T/E VI (E) cells. Ev - Empty vector.

WNT/β-catenin and TGF-β signaling pathways share key molecules (p38 MAPK, SNAI1/2, ZEB1/2 [16]) and can synergistically induce changes associated with EMT. Since we had identified an EMT transcriptional signature and upregulation of the WNT/β-catenin target genes in T/E overexpressing cells (Figure 2A–2C), we characterized signaling downstream of WNT in more detail. Overexpression of both T/E variants led to increased β-catenin signaling (Figure 6A), which was 2.4-fold higher in T/E VI compared to T/E III overexpressing cells. To test whether induction of gene expression was mediated by FZD4 upregulation, we incubated cells with rhFZD4. Disruption of FZD4-signaling showed a clear reduction of p38 phosphorylation in T/E III and T/E VI cells by Western blot (Figure 6B), and band quantification (Figure 6C). Further, we observed reduced *MMP1*, *VIM*, and *CDH2* levels in T/E III and VI (Figure 6D and 6E) and additionally reduced *SNAI2* in T/E VI cells (Figure 6E). These data suggest that FZD4-induced oncogenic effects of T/E overexpression are mediated by p38.

### Upregulation of *miR-503* in T/E VI cells promotes EMT by targeting *SMAD7*

**Figure 7:**
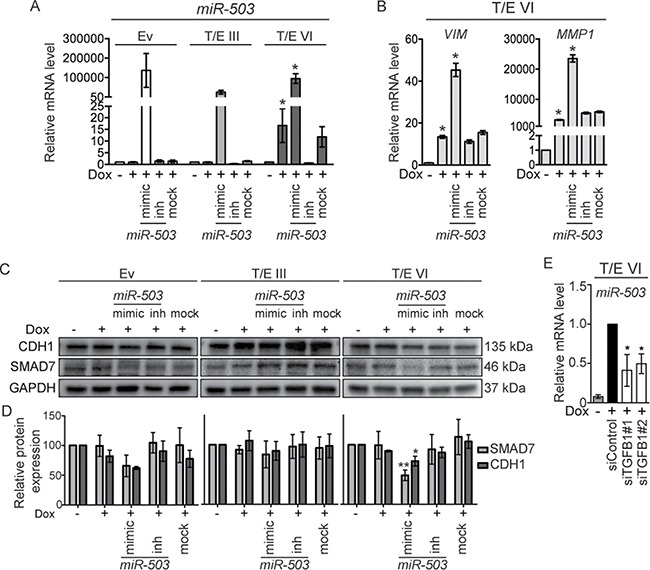
*MiR-503* overexpression in T/E VI cells inhibits SMAD7 and CDH1 **(A)** qPCR analysis of *miR-503* expression relative to *RNU6B*. Values are presented as mean ± s.d. of three independent experiments. **(B)** qPCR determination of *VIM* and *MMP1* expression in T/E VI expressing cells relative to *GAPDH*. **(C)** Western blot analysis of SMAD7 and CDH1 protein expression in T/E cells. Representative results of three independent Western blot experiments are shown. **(D)** Quantitative analysis of protein expression relative to GAPDH is presented as mean ± s.d. of three independent experiments. **(E)** *miR-503* expression upon siRNA-mediated knockdown of *TGFB1* (20 nM) in T/E VI cells was determined by qPCR. Mimic - hsa-*miR-503-5p* mimic (used at 10 nM), inh - hsa-*miR-503-5p* inhibitor (used at 100 nM).

We further aimed to identify determinants of the stronger activation of EMT regulating pathways genes in T/E VI expressing cells. We focused on *miR-503*, which was strongly upregulated in the *T/E VI only* (∼7-fold), but not in the *T/E III only* microarray dataset (Supplementary Table 1). A search for potential *miR-503* targets using *in silico* prediction algorithms showed that *miR-503* could target *SMAD7* [28], which was downregulated in T/E VI, but upregulated in T/E III cells. We therefore hypothesized that *miR-503* might be a candidate modulating the biological activity of the T/E VI fusion variants. To test whether *miR-503* could augment EMT, we transiently overexpressed and inhibited *miR-503* in T/E III and T/E VI cells using *miR-503* mimics and inhibitors, respectively. Only induced T/E VI cells displayed significant upregulation of *miR-503*, which was further increased by simultaneous *miR-503* overexpression (Figure 7A). Key EMT markers like *VIM* and *MMP1* were upregulated in T/E-induced cells and were further increased after *miR-503* overexpression (Figure 7B). Inhibition of *miR-503* upon induction of T/E VI expression led to a reduction of *VIM* (Figure 7B). The impacts of *miR-503* overexpression and inhibition on SMAD7, and CDH1 quantities as surrogate for a mesenchymal phenotype were examined by Western blot analysis. *MiR-503* overexpression led to decreased SMAD7 and CDH1 expression in T/E VI cells (Figure 7C and 7D). These results suggested that T/E VI-mediated overexpression of *miR-503* plays an important role in increasing EMT effectors and that *miR-503* can induce invasion of T/E VI expressing cells due to its ability to downregulate *CDH1*. Furthermore, *TGFB1* knockdown in T/E VI cells showed reduced expression of *miR-503* (Figure 7E), suggesting that the expression of *miR-503* is regulated by TGF-β, thereby contributing to enhanced TGF-β signaling by inhibition of *SMAD7* [29]. The varying SMAD7 levels are consistent with the observed differences in TGF-β and WNT/β-catenin signaling activity between T/E III and T/E VI cells.

## DISCUSSION

**Figure 8:**
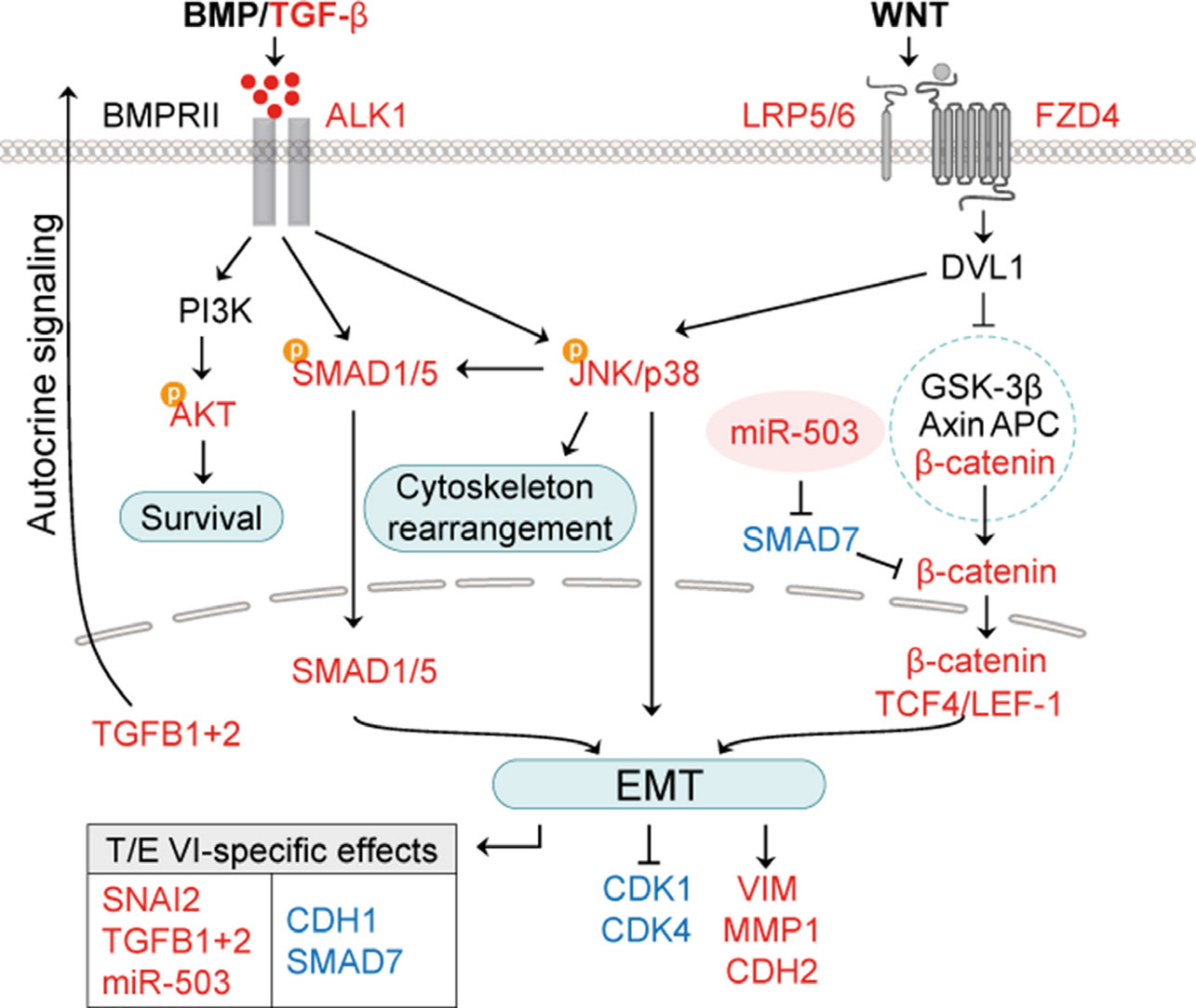
Working model for T/E-induced effects on BMP/TGF-β and WNT/β-catenin signaling pathways T/E VI variant-specific transcriptional modulation of *miR-503* and SMAD7 leads to stronger activation of EMT-regulating genes compared to T/E variant III. Red - upregulation; blue - downregulation.

Previously, we reported that transcriptional changes in T/E positive tumors are associated with deregulated TGF-β/BMP and WNT signaling pathways [13]. Here, by deploying inducible T/E overexpression in LNCaP cell models, we show that overexpression of two distinct T/E variants induce common as well as unique signaling programs that are able to lead the cells into EMT. Upon T/E overexpression, the cells acquire mesenchymal, fibroblast-like morphologies [30]. On the molecular level, this is accompanied by downregulation of AR, suggesting that ERG disrupts a lineage-specific differentiation program of prostate cells [18, 19], and upregulation of EMT effector genes, like *MMP1* and *VIM*, which are correlated with poor PCa tissue differentiation [31] and metastasis formation [32]. Furthermore, upregulated EMT-associated genes included the transcription factors *ZEB1* [30] and *TCF/LEF-1* [33], as well as *TGFB1* and *-2* [34]. We also show that T/E overexpression significantly enhances the invasion capability of LNCaP cells. These results are in agreement with the role of T/E overexpression in promoting cell invasion via induction of matrix metalloproteinase and plasminogen activator genes [3, 5, 12]. Global gene expression analysis of T/E overexpressing cells led to significantly overrepresented GO categories (proliferation and invasion), which correlated with the observed cellular phenotype. We further found many components of known signaling pathways, including JNK/p38 MAPKs, AKT and SMAD1/5, to be deregulated (Figure 8). Importantly, *TGFB1* was identified as a regulator gene of T/E-induced transcriptional changes, which again supports our previous *ex vivo* data [13]. Increased TGF-β expression has been shown to induce a tumor-promoting phenotype [35] facilitating metastatic dissemination [36]. TGF-β/BMP signaling is well known for its role in bone remodeling and metastasis formation in breast cancer [35] and could therefore play a role in promoting PCa metastases. Bone metastasis is a common site of PCa dissemination [37], and expression of TGF-β in PCa is correlated to metastasis and survival [38]. Intriguingly, serum TGF-β concentrations are elevated in PCa patients with bone metastases [39]. Furthermore, TGF-β protein and RNA expression was higher in bone metastases compared to visceral metastases in rapid autopsy specimens of patients who died of metastatic PCa [40] and was associated with a fibroblast-like phenotype [40]. We therefore propose that T/E-induced TGF-β secretion could have autocrine effects promoting tumor progression.

The T/E variants III and VI deploy two routes of ERG-mediated oncogenic pathway activation (Figure 8). The first route is characterized by strong upregulation of the TGF-β receptor ALK1, which can be activated by various BMPs, in addition to TGF-β1 and TGF-β3 [41, 42]. Inhibition of ALK1 in our model led to reduced phosphorylation of p38, downregulation of the EMT-markers *VIM*, *MMP1*, *CDH2*, and *SNAI2*, as well as reduced expression of *ID1* and *ID2*, for which induction by ALK1 signaling is known [43]. ALK1-induced expression of ID1 promotes tumor cell metastasis [44]. ID1 was shown to be involved in mesenchymal-to-epithelial transition (MET) of breast cancer cells during lung colonization after having undergone TGF-β-induced EMT [45]. High levels of ALK1 protein in tumor blood vessels can serve as a prognostic marker for metastatic disease in breast cancer patients [45]. In addition, the pharmacological inhibition of ALK1 was able to prevent metastatic dissemination and lung colonization in mouse models of endocrine pancreatic and mammary carcinomas [45, 46]. The high upregulation of ALK1 in T/E expressing cells in our study was accompanied by augmented levels of TGF-β1 and TGF-β2 mRNA and protein, suggesting that autocrine TGF-β signaling mediates ALK1 pathway activation and phenotypic cellular changes in T/E cells [47, 48].

The second route of T/E-induced signaling pointed towards FZD4 receptor-mediated WNT/β-catenin signaling as an important element [49, 50]. Evidence for the role of WNT signaling was seen in the upregulation of transcription factors of the T cell factor/lymphoid enhancer family (*TCF7L2*, *LEF1*), increased β-catenin reporter activity and downregulation of the negative regulator *CDH1* [51]. These effects were more profound in T/E VI, compared to T/E III, expressing cells. Importantly, *FZD4* was upregulated upon T/E overexpression, and inhibition of FZD4 led to reduced phosphorylation of p38. These results confirmed reports that loss of cell adhesion and EMT were associated with FZD4-induced activation of WNT signaling [49].

Our results further suggest that variant-specific transcriptional modulation is responsible for the differences in activation of EMT regulating pathway genes. Strikingly, we observed upregulation of *miR-503* exclusively in T/E VI overexpressing cells. Overexpression of *miR-503* was able to repress expression of SMAD7, a known negative regulator of TGF-β and WNT/β-catenin signaling [52]. Thus, the *miR-503*-mediated downregulation of *SMAD7* in T/E VI, but not in T/E III cells, explains T/E VI variant-specific transcription. Recently, Li et al. could show that *miR-503* downregulates SMAD7 expression and thereby enhances TGF-β signaling and the metastatic capability of breast cancer cells [28]. SMAD7-mediated stabilization of β-catenin binding to E-cadherin turned out to increase cell-cell adhesion and formation of adherens junctions [53], thereby potentially blocking metastasis. Reduced expression of SMAD7 might account for the stronger increase of TGF-β signaling and β-catenin reporter activity observed in T/E VI cells. Zhu et al. showed that stimulation of fibroblasts with recombinant TGF-β results in a decreased expression of SMAD7 [54]. In agreement with previous reports in MCF-10A breast cancer cells [55], *TGFB1* knockdown also decreased *miR-503* expression. *MiR-503*-mediated repression of *SMAD7* therefore appears to be a way to escape the inhibitory effect of SMAD7 on TGF-β and WNT/β-catenin signaling. Although *miR-503* expression was shown to be lower in metastatic compared to non-metastatic PCa xenografts [56], and several studies reported tumor suppressor properties of *miR-503* [57, 58], in the context of T/E-induced TGF-β signaling *miR-503* overexpression has tumor-promoting effects.

In conclusion, our study identifies the TGF-β/BMP and WNT/β-catenin signaling pathways as molecular determinants underlying T/E-mediated EMT in PCa cells (Figure 8). We confirm that WNT/β-catenin signaling in T/E cells is mediated by FZD4 and propose that *miR-503* plays a crucial role in augmenting this process. We further demonstrate that TGF-β-ALK1-p38 signaling promotes EMT in T/E expressing cells. Our findings suggest that autocrine activation of ALK1 plays a role in PCa cells. This could provide a rational basis for ALK1-blocking agents (which are currently already tested in clinical studies in various malignancies [59, 60]) to inhibit progression of ERG-positive PCa.

## MATERIALS AND METHODS

### Cell lines and culturing

LNCaP (CRL-1740) and NCI-H660 (CRL-5813) cells were purchased from American Type Culture Collection (ATCC, Manassas, VA, USA). Stably transfected acceptor LNCaP cells were maintained in RPMI1640 (Thermo Fisher Scientific, Waltham, MA, USA), supplemented with 10% of Tet System Approved FBS (tet-FBS, Clontech, Göteborg, Sweden) and 80 μg/mL hygromycin B (Thermo Fisher Scientific). NCI-H660 cells were maintained in HITES medium supplemented with 5% fetal bovine serum according to the provider's instructions. All cell lines were authenticated using Multiplex Cell Authentication by Multiplexion (Heidelberg, Germany) as described recently [61]. The SNP profiles matched known profiles or were unique. The purity of cell lines was validated using the Multiplex cell Contamination Test by Multiplexion (Heidelberg, Germany) as described recently [62]. No Mycoplasma, SMRV or interspecies contamination was detected.

### Generation of LNCaP cell models stably expressing T/E variants

Establishment of the LNCaP-T/E variant cell model including T/E sequences is described in the Supplementary Methods (Supplementary Figure 1A and Supplementary Figure 2). Transgene expression was induced with 50 ng/mL Dox (Sigma-Aldrich, Munich, Germany) in RPMI1640 containing 10% tet-FBS. Medium of the uninduced cells was supplemented with the respective volume of PBS only.

### RNA isolation, reverse transcription and quantitative real-time PCR

Total RNA was isolated from cell lines using the miRNeasy Mini Kit (Qiagen, Hilden, Germany) and quality controlled on the 2100 Bioanalyzer (Agilent Technologies, Waldbronn, Germany) with RNA 6000 Nano Kit according to manufacturer's protocols. Total RNA was reverse transcribed using the RevertAid H Minus First Strand cDNA Synthesis Kit (Thermo Fisher Scientific). HotStar*Taq*DNA polymerase (Qiagen) was used for RT-PCR with 50 ng of cDNA template. Relative mRNA levels were assessed by quantitative RT-PCR on the Lightcycler 480 (Roche Diagnostics, Mannheim, Germany) using Universal Probe Library (UPL) assays and primers listed in Supplementary Table 5. Linear expression levels were normalized to *GAPDH* using the 2^(−ΔΔCt)^ method [63]. For miRNA quantification, TaqMan^®^ Assays (*hsa-miR-503*, ID: 1048; *RNU6B*, ID: 1093, Thermo Fisher Scientific) were used according to the manufacturer's instruction.

### Microarray gene expression profiling

RNA was isolated with the RNase-Free DNase Set (Qiagen) according to the manufacturer's protocol. After quality control, 500 ng of total RNA with a concentration of 50 ng/μl were submitted to the DKFZ Genomics and Proteomics Core Facility (GPCF) for Illumina Whole-Genome Expression Beadchip Analysis (Human HT-12 Chip). The raw data were quantile-normalized using the Bioconductor package *preprocessCore* in R. The microarray data reported in this study are available from the NCBI GEO database (GSE78032). Genes showing expression fold change > 1.5 (*p-value* < 0.05) were considered as differentially expressed and were analyzed with Ingenuity Pathway Analysis (IPA) (see below). Genes involved in relevant biological processes obtained from microarray analysis were validated by qPCR in the same samples that were used for microarray profiling.

### Luciferase reporter assay

Cells seeded in triplicate in 96-well plates at 5000 cells/well were treated with Dox and transfected with 100 ng of either wild-type TOPflash or mutant FOPflash reporter plasmid from the TCF Reporter Plasmid Kit (Merck Millipore, Darmstadt, Germany) using the JetPei Polyplus transfection reagent (VWR International, Darmstadt, Germany). Firefly luciferase signals were determined 72 h after transfection using the ONE-Glo^TM^ Luciferase Assay System (Promega, Mannheim, Germany). Fold activation of WNT/β-catenin pathway was calculated by dividing wild-type TOPflash by mutant FOPflash activity.

### siRNA-mediated gene knock-down

LNCaP-T/E cells were transfected with 20nM siRNA against *TGFB1* (Qiagen) using Lipofectamine RNAiMAX (Thermo Fisher Scientific) and OptiMEM^®^ I (Thermo Fisher Scientific) according to the manufacturer's protocol. Cells transfected with nonsilencing AllStars Negative Control siRNA (Qiagen) were used as controls. Cells were treated for 48 h, medium was changed, Dox-supplemented medium was added where indicated and siRNA treatment was repeated. Cells were incubated for 72 h and processed for further analysis.

NCI-H660 cells were transfected with 50nM siRNA against *ERG* (Qiagen) using Lipofectamine RNAiMAX (Thermo Fisher Scientific) and OptiMEM^®^ I (Thermo Fisher Scientific) according to the manufacturer's protocol. Cells transfected with nonsilencing AllStars Negative Control siRNA (Qiagen) were used as controls. Cells were incubated for 72 h and processed for further analysis.

### miRNA transfection

For transfection, cells were treated with Dox and transfected with *hsa-miR-503-5p* inhibitor (Exiqon, Vedbaek, Denmark) or *hsa-miR-503-5p* mimic (GE Healthcare, Rosersberg, Sweden) using Lipofectamine RNAiMAX transfection reagent (Thermo Fisher Scientific).

### Pharmacological inhibitors

The ALK1 inhibitor K02288 (Biomol, Hamburg, Germany) was dissolved at a concentration of 100 mM in DMSO. Further dilutions of K02288 were made in PBS to reduce the final concentration of DMSO in the assay. Equal amounts of DMSO added to the cell culture medium served as negative control. RhALK1 (R&D, Wiesbaden, Germany) was dissolved in PBS at a concentration of 100μg/mL, and rhFZD4 (R&D) was dissolved in PBS at a concentration of 400 μg/mL. Here, PBS was added to the cell culture medium as a negative control. Inhibitors or control solvents were diluted in Dox-containing tet-FBS medium and added to the cells for 48h.

### Cell proliferation assay

Cells were treated with Dox for 48 h and seeded into 96-well plates at 5000 cells/well in 90 μl 10% tet-FBS-containing medium in triplicate. Ten μl of the colorimetric WST-1 reagent (Roche Diagnostics) was added to the medium and incubated at 37°C at the indicated time points. Absorbance was measured one hour after addition of WST-1 reagent using a Tecan Infinite^®^ M200 microplate reader (Tecan Group Ltd., Männedorf, Switzerland).

### Migration and invasion assays

*In vitro* cell migration assays were performed in duplicate using 24-well transwell chambers with 8 μm pore size (Merck Millipore). Cells (5 × 10^5^ cells/ml) were seeded in the upper chamber in 200 μl serum-free medium. 700 μl of medium supplemented with 10% FBS as chemoattractant was filled into the bottom well. After 48 h of cultivation in 5% CO_2_ at 37°C, migrated cells attached to the lower surface of the insert were fixed with 100% methanol on ice and stained with 0.1% Crystal Violet (Sigma- Aldrich). Migrated cells were counted in four random fields under a light microscope (10× magnification).

Invasion assays were performed analogously after coating the transwell chambers with 100 μl Matrigel (BD Biosciences, Heidelberg, Germany) per filter.

### Cell cycle analysis

Twenty-four, 48 and 72 h after Dox induction, trypsinized cells were fixed with 100% ice-cold ethanol and stained with propidium iodide (PI) solution (PBS containing 50 μg/ml PI, 0.1mg/ml RNase A, 0.05% (v/v) Triton X-100). PI staining was analyzed using a FACSCanto II flow cytometer (BD Biosciences). Data was analyzed using the software Cyflogig, version 1.2.1 (CyFlo Ltd., Turku, Finland).

### Cell lysis and western blot analysis

Whole-cell lysates were prepared in RIPA lysis buffer (50 mM Tris-HCl pH 8.0, 150mM NaCl, 1% NP-40, 0.5% sodium deoxycholate, 0.1% SDS), supplemented with 1× cOmplete Mini Protease Inhibitor Cocktail (Roche Diagnostics) and 1× PhosSTOP Phosphatase Inhibitor Cocktail (Roche Diagnostics). Lysates were boiled 5 min at 95°C with 4× reducing Roti-Load protein loading buffer (Roth, Karlsruhe, Germany). Samples were separated on a mini polyacrylamide gel (Bio-Rad, Munich, Germany) and transferred to PVDF membranes using the Trans-Blot Turbo semi-dry blotting system (Bio-Rad) at 1.3A, 25V for 7–10 min. After blocking with 5% BSA in Tween-20/PBS, membranes were probed with primary antibodies prepared in blocking solution overnight at 4°C on a roller, followed by incubation with horseradish peroxidase-conjugated secondary antibody in blocking solution for 1 h at room temperature and ECL detection (Thermo Fisher Scientific) by the ChemiDoc XRS+ system (Bio-Rad). Western blotting was performed using primary antibodies against ERG (Abcam, Cambridge, UK), and SMAD7 (Abcam), AR (Santa Cruz, Dallas, Texas, USA), p-SMAD1/5, SMAD1, p-p38, p38, p-AKT, AKT, CDH1, and GAPDH (all Cell Signaling Technology, Danvers, MA, USA) at 1:1000 dilution. Secondary antibodies used were anti-rabbit-HRP (at 1:25000 dilution; Dianova, Hamburg, Germany) and anti-mouse-HRP (at 1:10000 dilution; Cell Signaling). Quantitative analysis of protein expression relative to GAPDH was done using Image Lab software (Bio-Rad).

### Luminex immunoassay

TGF-β signaling pathway components were analyzed using the Milliplex Human TGF-β signaling 6-plex (Merck Millipore) and the Milliplex Human Multi-pathway 9-plex assay (Merck Millipore). Total TGF-β protein expression in cell lysates and cell culture supernatants were measured using the Milliplex Human TGF-β 1,2,3 assay (Merck Millipore). For Luminex analysis, the cells were treated with Dox to induce ERG expression. For collection, cell lysates and supernatants were centrifuged (15 min, 4000 rpm, 4°C) after 72 h of induction. Immunoassays using Luminex^®^ xMAP^®^ technology were performed according to the manufacturer's instructions. The fluorescent reporter signals were analyzed by a Bio-Plex 200 reader (Bio-Rad).

### Ingenuity pathway analysis

Functional annotation and pathway enrichment of differentially regulated genes were identified using Ingenuity Pathway Analysis (IPA) software (Qiagen). IPA uses the Ingenuity knowledge base, a database of protein and gene interactions integrated from published biomedical literature and 3rd party sources. Analysis using IPA was performed between December 2015 and February 2016 (Ingenuity version 26127183). Genes showing expression fold change > 1.5 were considered as differentially expressed and included in the analysis.

### Functional annotations

Gene expression changes were categorized into functional annotations of molecular and cellular mechanisms. The Ingenuity knowledge base provides a predicted direction of change for the biological function (downstream effect analysis), represented by an activation z-score, where z > 2.0 or < −2.0 is predictive for activation or reduction of the process, respectively. A *p-value* < 0.05 indicates a statistically significant association between a set of differentially expressed genes and a given process.

### Pathway enrichment analysis

Ingenuity knowledge base provides an analysis of metabolic and cell signaling pathways that are significantly enriched in the gene expression signature. Pathway significance values were calculated based on Fisher's right tailed exact test and the –log(*p-value*) by IPA. Pathways meeting the threshold *p-value* < 0.05 were considered as significant. Using the ‘Compare’ tool, IPA identified the intersection and unique gene sets among T/E III and T/E VI versus empty vector datasets. Upstream regulator analysis can identify molecules upstream of the genes in the dataset that potentially explain the observed gene expression changes and molecular functions. It is based on prior knowledge of expected effects between transcriptional regulators and their target genes stored in the Ingenuity knowledge base.

### Statistical testing

Expression differences between the induced and uninduced cells were analyzed using a paired *t-test* and between induced cells of T/E III and VI by an unpaired *t-test*. Statistical significance of *t-test* depicted as *p < 0.05, **p < 0.01, ***p < 0.001.

## SUPPLEMENTARY MATERIALS AND METHODS FIGURES AND TABLES









## Abbreviations

ACPP: Acid phosphatase, prostate
AKT: AKT serine/threonine kinase 1
ALK1: Activin receptor-like kinase 1
AR: Androgen receptor
BAMBI: BMP and activin membrane-bound inhibitor
BMP: Bone morphogenetic protein
CD24: Cluster of differentiation 24
CDH1: E-cadherin
CDH2: N-cadherin
CDK1: Cyclin-dependent kinase 1
Dox: Doxycycline
EMT: Epithelial-to-mesenchymal transition
ERG: V-ets erythroblastosis virus E26 homolog (avian)
ERK2: Extracellular signal-regulated kinases, alias of MAPK1
Ev: Empty vector
FN1: Fibronectin 1
FZD4: Frizzled 4
GAPDH: Glyceraldehyde-3-phosphate dehydrogenase
GO: Gene Ontology
ID1 and 2: Inhibitor of differentiation (1 and 2)
IPA: Ingenuity Pathway Analysis
JNK: c-Jun N-terminal kinases
LRP5 and 6: Low-density lipoprotein receptor-related protein 5 and 6
MAPK: Mitogen-activated protein kinase
MET: Mesenchymal-to-epithelial transition
miR: micro-RNA
MMP: Matrix metalloproteinase
MSMB: Microseminoprotein beta
P38: alias of MAPK14, mitogen-activated protein kinase 14
pAKT: phospho-AKT
PCa: Prostate cancer
PI3K: Phosphatidylinositol 3-kinase
PLA1A: Phospholipase A1 member A
PLAT: Plasminogen activator, tissue type
p-p38: phospho-p38
pSMAD: phospho-SMAD
qPCR: quantitative reverse transcription PCR
rhALK1: recombinant decoy receptor ALK1
rhFZD4: recombinant decoy receptor FZD4
RT-PCR: Reverse transcription PCR
siRNA: small interfering RNA
SLC45A3: Solute carrier family 45 member 3
SMAD: SMAD family protein
SNAI2: Snail family transcriptional repressor 2
TCF7L2: T cell factor/lymphoid enhancer 2
TCF/LEF-1: Transcription factor/lymphoid enhancer binding factor 1
TDRD1: Tudor domain containing 1
T/E: TMPRSS2:ERG
TGF-β: Transforming growth factor beta
TGFB1 and 2: TGF-β 1 and 2
TMPRSS2: Transmembrane protease, serine 2
VIM: Vimentin
VTN: Vitronectin
WNT: Wingless-type family member
ZEB1: Zinc finger E-box binding homeobox 1
