# Supplementary material for: TMPRSS2:ERG gene fusion variants induce TGF-β signaling and epithelial to mesenchymal transition in human prostate cancer cells
Source: Oncotarget. 2017 Mar 6;8(15):25115–30. doi: 10.18632/oncotarget.15931 (PMC5421914; doi:10.18632/oncotarget.15931)
Supplement: Supplementary file 1 [file oncotarget-08-25115-s001.pdf]

TMPRSS2:ERG gene fusion variants induce TGF-β signaling and epithelial to mesenchymal transition in human prostate cancer cells

Supplementary Materials

SUPPLEMENTARY METHODS

Generation of LNCaP cell models stably expressing T/E variants

First, cDNA fragments of the two *TMPRSS2:ERG* variants were amplified by RT-PCR from the ERG ORF plasmid (RefSeq NM_004449.4) (provided by the DKFZ Genomics and Proteomics Core Facility (GPCF)) and cloned into a Gateway entry vector using BP Clonase Enzyme Mix (Thermo Fisher Scientific, Waltham, MA, USA). Sequences of cloning primer pairs are listed in Supplementary Table 5. DNA fragments were transferred from the entry vector to an expression vector carrying the tet repressor gene (tetR) as well as a doxycycline (Dox)-regulated CMV/Tet operator (TetO) promoter by LR Clonase Mix (Thermo Fisher Scientific). In a second step, the LNCaP acceptor clone #126 was generated by transfection with a vector carrying a Flp recombinase target (FRT) site. The single integration of the vector was verified by Southern blot hybridization, and the cells were selected for successful integration using neomycin resistance and green fluorescence as markers (Supplementary Figure 1A). Finally, LNCaP#126 cells were co-transfected with a plasmid encoding the Flp recombinase, and the expression vector construct carrying a FRT site and the different target genes under control of the Dox-regulated CMV/Tet operator (TetO) promoter. A HcRed expression construct served as proof for successive integration of transgenes into LNCaP#126 (Supplementary Figure 1A) by showing that upon disruption of the EGFP ORF green fluorescence was abolished. Induction of transgene expression by Dox for 72 h activated the red fluorescence reporter gene in this case, compared to the uninduced state. Subsequently, the target T/E sequence variants III and VI (Supplementary Figure 2), respectively, under control of the Dox-regulated CMV/Tet operator (TetO) promoter were integrated into the LNCaP#126 cells in the same manner (LNCaP-T/E cells).

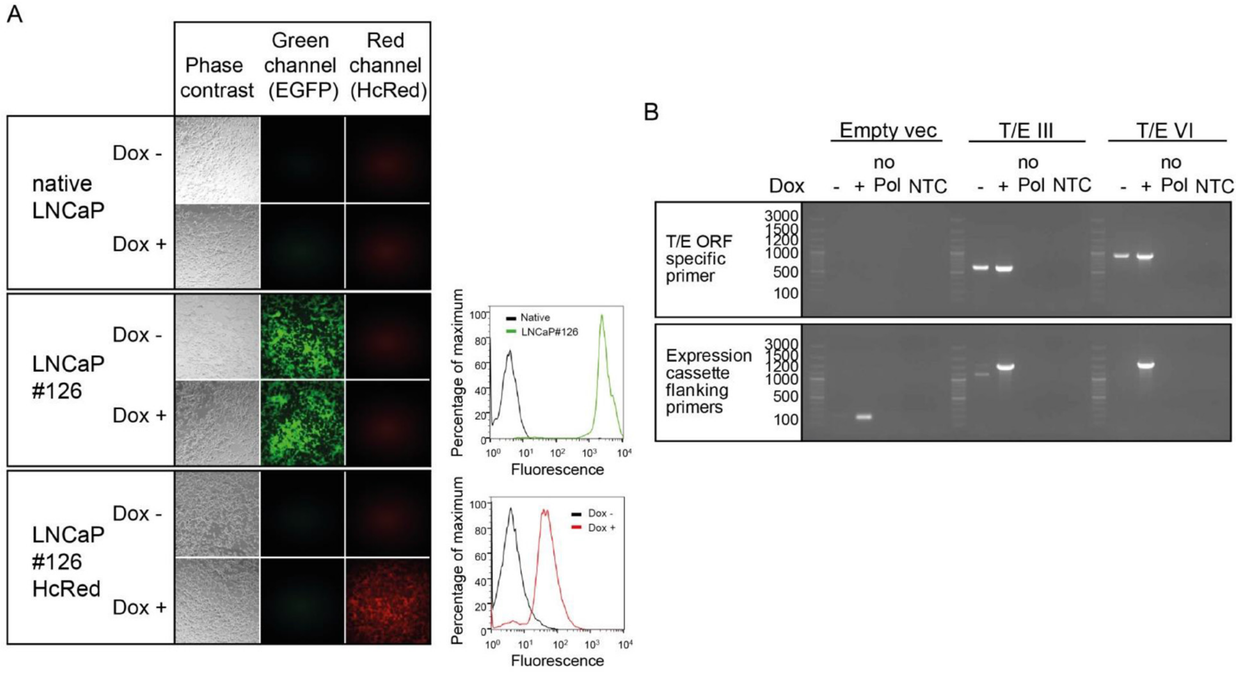

Supplementary Figure 1: Site-specific recombination-based system for generation of stably transfected LNCaP cells. (**A**) Fluorescence microscopy (left) and FACS analysis (right) of recombination events in LNCaP cells. Native LNCaP cells showing no fluorescence in both, green and red, channels (top left panel). LNCaP acceptor clone #126 after selection for neomycin resistance and green fluorescence: LNCaP cells successfully integrated the acceptor plasmid leading to expression of EGFP independent of Dox (left middle panel and upper right panel). LNCaP cells stably recombined with a HcRed expression plasmid after selection for hygromycin resistance. Successful recombination and integration of the expression plasmid disrupted the EGFP ORF and abolished green fluorescence of the LNCaP acceptor clone. Induction of transgene expression by Dox for 72 h activated the red fluorescence reporter gene, compared to the uninduced state (left bottom panel). Fluorescence signal after induction depicts a homogeneous expression throughout the whole cell population (bottom right). Microscopic pictures were taken at 10-fold magnification. (**B**) Agarose gel electrophoresis of RT-PCR products after *ERG* overexpression in LNCaP empty vector and T/E clones III and VI, respectively. RT-PCR products from amplification with T/E specific primers for integrated sequences (upper panel) and from amplification with flanking primers for the complete ORF including parts of the expression cassette (lower panel) from induced (+) and uninduced (−) cells. Expected sizes of T/E specific PCR products are 599bp, and 866bp for the T/E inserts III and VI, respectively. Expected sizes of PCR products from flanking primers are 1481bp, 1559bp and 209bp for T/E III, VI and the empty vector, respectively. no Pol: no Polymerase negative control, NTC: no template negative control.

T/E III

ATGACCGCGTCCTCCTCCAGCGACTATGGACAGACTTCCAAGATGAGCCCACGCGTCCCTCAGCAGGATTG
GCTGTCTCAACCCCCAGCCAGGGTCACCATCAAAATGGAATGTAACCCTAGCCAGGTGAATGGCTCAAGGA
ACTCTCCTGATGAATGCAGTGTGGCCAAAGGCGGGAAGATGGTGGGCAGCCCAGACACCGTTGGGATGAAC
TACGGCAGCTACATGGAGGAGAAGCACATGCCACCCCCAAACATGACCACGAACGAGCGCAGAGTTATCGT
GCCAGCAGATCCTACGCTATGGAGTACAGACCATGTGCGGCAGTGGCTGGAGTGGGCGGTGAAAGAATATG
GCCTTCCAGACGTCAACATCTTGTTATTCCAGAACATCGATGGGAAGGAACTGTGCAAGATGACCAAGGAC
GACTTCCAGAGGCTCACCCCCAGCTACAATGCCGACATCCTTCTCTCACATCTCCACTACCTCAGAGAGAC
TCCTCTTCCACATTTGACTTCAGATGATGTTGATAAAGCCTTACAAAACTCTCCACGGTTAATGCATGCTA
GAAACACAGATTTACCATATGAGCCCCCCAGGAGATCAGCCTGGACCGGTCACGGCCACCCCACGCCCCAG
TCGAAAGCTGCTCAACCATCTCCTTCCACAGTGCCCAAAACTGAAGACCAGCGTCCTCAGTTAGATCCTTA
TCAGATTCTTGGACCAACAAGTAGCCGCCTTGCAAATCCAGGCAGTGGCCAGATCCAGCTTTGGCAGTTCC
TCCTGGAGCTCCTGTCGGACAGCTCCAACTCCAGCTGCATCACCTGGGAAGGCACCAACGGGGAGTTCAAG
ATGACGGATCCCGACGAGGTGGCCCGGCGCTGGGGAGAGCGGAAGAGCAAACCCAACATGAACTACGATAA
GCTCAGCCGCGCCCTCCGTTACTACTATGACAAGAACATCATGACCAAGGTCCATGGGAAGCGCTACGCCT
ACAAGTTCGACTTCCACGGGATCGCCCAGGCCCTCCAGCCCCACCCCCCGGAGTCATCTCTGTACAAGTAC
CCCTCAGACCTCCCGTACATGGGCTCCTATCACGCCCACCCACAGAAGATGAACTTTGTGGCGCCCCACCC
TCCAGCCCTCCCCGTGACATCTTCCAGTTTTTTTGCTGCCCCAAACCCATACTGGAATTCACCAACTGGGG
GTATATACCCCAACACTAGGCTCCCCACCAGCCATATGCCTTCTCATCTGGGCACTTACTACTAA

T/E VI

ATGGCTTTGAACTCAGAAGCCTTATCAGTTGTGAGTGAGGACCAGTCGTTGTTTGAGTGTGCCTACGGAAC
GCCACACCTGGCTAAGACAGAGATGACCGCGTCCTCCTCCAGCGACTATGGACAGACTTCCAAGATGAGCC
CACGCGTCCCTCAGCAGGATTGGCTGTCTCAACCCCCAGCCAGGGTCACCATCAAAATGGAATGTAACCCT
AGCCAGGTGAATGGCTCAAGGAACTCTCCTGATGAATGCAGTGTGGCCAAAGGCGGGAAGATGGTGGGCAG
CCCAGACACCGTTGGGATGAACTACGGCAGCTACATGGAGGAGAAGCACATGCCACCCCCAAACATGACCA
CGAACGAGCGCAGAGTTATCGTGCCAGCAGATCCTACGCTATGGAGTACAGACCATGTGCGGCAGTGGCTG
GAGTGGGCGGTGAAAGAATATGGCCTTCCAGACGTCAACATCTTGTTATTCCAGAACATCGATGGGAAGGA
ACTGTGCAAGATGACCAAGGACGACTTCCAGAGGCTCACCCCCAGCTACAATGCCGACATCCTTCTCTCAC
ATCTCCACTACCTCAGAGAGACTCCTCTTCCACATTTGACTTCAGATGATGTTGATAAAGCCTTACAAAAC
TCTCCACGGTTAATGCATGCTAGAAACACAGATTTACCATATGAGCCCCCCAGGAGATCAGCCTGGACCGG
TCACGGCCACCCCACGCCCCAGTCGAAAGCTGCTCAACCATCTCCTTCCACAGTGCCCAAAACTGAAGACC
AGCGTCCTCAGTTAGATCCTTATCAGATTCTTGGACCAACAAGTAGCCGCCTTGCAAATCCAGGCAGTGGC
CAGATCCAGCTTTGGCAGTTCCTCCTGGAGCTCCTGTCGGACAGCTCCAACTCCAGCTGCATCACCTGGGA
AGGCACCAACGGGGAGTTCAAGATGACGGATCCCGACGAGGTGGCCCGGCGCTGGGGAGAGCGGAAGAGCA
AACCCAACATGAACTACGATAAGCTCAGCCGCGCCCTCCGTTACTACTATGACAAGAACATCATGACCAAG
GTCCATGGGAAGCGCTACGCCTACAAGTTCGACTTCCACGGGATCGCCCAGGCCCTCCAGCCCCACCCCCC
GGAGTCATCTCTGTACAAGTACCCCTCAGACCTCCCGTACATGGGCTCCTATCACGCCCACCCACAGAAGA
TGAACTTTGTGGCGCCCCACCCTCCAGCCCTCCCCGTGACATCTTCCAGTTTTTTTGCTGCCCCAAACCCA
TACTGGAATTCACCAACTGGGGGTATATACCCCAACACTAGGCTCCCCACCAGCCATATGCCTTCTCATCT
GGGCACTTACTACTAA

Supplementary Figure 2: T/E sequences cloned into the expression vector. Underlined: sequence coding for the first five amino acids of TMPRSS2 in T/E VI.

Supplementary Table 1: List of genes with expression fold change > 1.5 that were used in IPA. See Supplementary_Table_1

Supplementary Table 2: Functional annotation of genes differentially regulated upon T/E overexpression

Functional annotation	Genes	*p*-value	z-score*	#Genes†
Proliferation of cells	*SET, ATP5G1, STMN1, LILRB1, MYC, EIF4A1, ACTN4, S100P, LCP1, SCL19A1*	1.63E-34	−2.545	768
Cell proliferation of tumor cell lines	*CD24, IL24, IGFBP3, SLPI, GJA1, PLAT, SH2D3C, ZFP36, BCL6, SGK1*	9.02E-28	−1.640	372
Interphase	*MYC, FBXO5, CDKN1B, ERBB2, E2F2, PNPT1, BRCA1, CCNE1, BIRC5, CDKN3*	4.10E-19	−2.287	173
Invasion of cells	*CD24, CTNNAL1, GJA1, PLAT, TFF1, CTSK, SHC1, S100A9, MMP1, ETV6*	3.23E-13	1.316	197
Survival of organism	*PTGER4, PLAT, BCL6, ID2, MCL1, BCL2L1, CDKN2A, TRAF3IP2, LMNA, NOS3*	8.51E-05	1.540	130

Top 10 differentially expressed genes in our dataset that were annotated to a function. A gene was selected when its annotation to the indicated function was based on at least two findings in the Ingenuity knowledge base. *Activation z-score is a measure of predicted change (increase or decrease) of the process. †Total number of genes supporting a specific functional annotation.

Supplementary Table 3: Canonical pathway analysis of genes differentially regulated upon T/E overexpression

Ingenuity Canonical Pathways	*p*-value	z-score*	#Genes†
Cell Cycle: G2/M DNA Damage Checkpoint Regulation	1.02E-07	1.886	19 (49)
Molecular Mechanisms of Cancer	3.79E-06	NaN	65 (365)
Estrogen-mediated S-phase Entry	7.59E-06	−2.53	11 (24)
Sertoli Cell-Sertoli Cell Junction Signaling	1.51E-05	NaN	37 (178)
Mitotic Roles of Polo-Like Kinase	1.75E-05	−2.138	19 (66)

Significantly enriched canonical pathways across the dataset of commonly regulated genes between T/E III and VI are shown. *Activation z-score is a measure of predicted change (activated or reduced) of the process. NaN – not a number. †Number of genes in the dataset, which are represented in the pathway. Numbers in brackets depict the total number of genes in the pathway in the reference gene set.

Supplementary Table 4: Genes predicted to be regulated by TGF-β according to IPA. See Supplementary_Table_4

Supplementary Table 5: List of primer sequences. See Supplementary_Table_5
