# Supplementary material for: TMPRSS2:ERG gene fusion variants induce TGF-β signaling and epithelial to mesenchymal transition in human prostate cancer cells
Source: Oncotarget. 2017 Mar 6;8(15):25115–30. doi: 10.18632/oncotarget.15931 (PMC5421914; doi:10.18632/oncotarget.15931)
Supplement: Supplementary file 4 [file oncotarget-08-25115-s004.docx]

**Table S5:** **List of primer sequences.**

**List of primers used for cloning**

**Name Sequence 5’ - 3’**

attB1_universal ggggacaagtttgtacaaaaaagcaggctccaccatg

attB2_universal ggggaccactttgtacaagaaagctgggtc

ERG_T/E_III_For ctcagcaggattggctgtct

ERG_T/E_III_Rev tggttgagcagctttcgact

ERG_T/E_VI_For gtgagtgaggaccagtcgtt

ERG_T/E_VI_Rev tgatgcagctggagttggag

**List of primers used for RT-PCR**

**Name Sequence 5’ - 3’**

T/E_III_for ctcagcaggattggctgtct

T/E_III_rev tggttgagcagctttcgact

T/E VI_for gtgagtgaggaccagtcgtt

T/E VI_rev tgatgcagctggagttggag

vec_empty_for gttttgacctccatagaagacac

vec_universal_rev caacagatggctggcaacta

**List of primers and probes used for qPCR**

**Name #UPL probe Sequence forward 5’ - 3’ Sequence reverse 5’ – 3’**

*ACVRL1* 71 agacccccaccatcccta cgcatcatctgagctaggc

*BAMBI* 71 cgccactccagctacatctt cacagtagcatcgaatttcacc

*BMP1* 18 ttcaaggcccacttcttctc cataactgccgaacgtgttg

*CCND1* 17 gctgtgcatctacaccgaca ttgagcttgttcaccaggag

*CCND2* 49 ggacatccaaccctacatgc cgcacttctgttcctcacag

*CDH1* 84 tggaggaattcttgctttgc cgctctcctccgaagaaac

*CDH2* 59 tcaacaatgagactggtgacatc tatgtgggattgccttccat

*CDK1* 79 tggatctgaagaaatacttggattcta caatcccctgtaggatttgg

*CDK4* 25 gtgcagtcggtggtacctg ttcgcttgtgtgggttaaaa

*DKK4* 37 aggaggtgccagcgagat tgcatcttccatcgtagtacaaa

*ERG_T/E* 64 ggttaatgcatgctagaaacaca agatggttgagcagctttcg

*FZD4* 19 aactttcacaccgctcatcc tgcacattggcacataaaca

*GADPH* 60 agccacatcgctcagacac gcccaatacgaccaaatcc

*ID1* 39 ccagaaccgcaaggtgag ggtccctgatgtagtcgatga

*ID2* 5 atatcagcatcctgtccttgc aaagaaatcatgaacaccgctta

*LEF1* 79 agatcaccccacctcttgg atgagggatgccagttgtgt

*MMP1* 26 acgaatttgccgacagagat gtccttggggtatccgtgta

*MMP1* 7 gctaacctttgatgctataactacga tttgtgcgcatgtagaatctg

*MMP10* 61 gcaaaagaggaggactccaa tcacatccttttcgaggttgta

*SMAD7* 69 acccgatggattttctcaaa aggggccagataattcgttc

*SNAI2* 73 acagcgaactggacacacat gatggggctgtatgctcct

*TCF7L2* 10 acgcctcttatcacgtacagc gggtaatacggggatatatctgg

*TGFB1* 72 gcagcacgtggagctgta cagccggttgctgaggta

*TGFB2* 67 ccaaagggtacaatgccaac cagatgcttctggatttatggtatt

*TGFBR3* 82 gatttcatcttcggcttgaaa gctcaggaggaatagtgtgga

*VIM* 13 tacaggaagctgctggaagg accagagggagtgaatccag

*ZEB1* 3 cagtgaaagagaagggaatgct cttcaggccccaggattt
